# Correction: Characterisation of intergrowth in metal oxide materials using structure-mining: the case of γ-MnO_2_

**DOI:** 10.1039/d3dt90071a

**Published:** 2023-04-27

**Authors:** Nicolas P. L. Magnard, Andy S. Anker, Olivia Aalling-Frederiksen, Andrea Kirsch, Kirsten M. Ø. Jensen

**Affiliations:** a Department of Chemistry and Nano-Science Center, University of Copenhagen 2100 Copenhagen Ø Denmark kirsten@chem.ku.dk

## Abstract

Correction for ‘Characterisation of intergrowth in metal oxide materials using structure-mining: the case of γ-MnO_2_’ by Nicolas P. L. Magnard *et al.*, *Dalton Trans.*, 2022, **51**, 17150–17161, https://doi.org/10.1039/D2DT02153F.

The authors regret that ref. 51 was incorrectly cited. The correct citation for ref. 51 is given below as ref. 1.

The Royal Society of Chemistry apologises for these errors and any consequent inconvenience to authors and readers.

## Supplementary Material
